# “The Buoy”: Utilization of a low-threshold ambulatory setting for traumatized children and adolescents in Austria

**DOI:** 10.1007/s40211-016-0178-x

**Published:** 2016-03-09

**Authors:** J. Huemer, S. Völkl-Kernstock, A. Yee, T. Bruckner, Katrin Skala

**Affiliations:** 1 Department of Child and Adolescent Psychiatry, Medical University of Vienna, Waehringer Guertel 18–20, 1090 Vienna, Austria; 2 Department of Psychiatry & Behavioral Sciences, Stanford University School of Medicine, Stanford, USA

**Keywords:** Child, Adolescent, Trauma, Outpatient, Utilization, Kind, Jugendliche, Trauma, Ambulant, Inanspruchnahme

## Abstract

**Background:**

This investigation intended to assess the use of an outpatient clinic providing low-threshold, short-term trauma therapy for children and adolescents across the first 6 years of its existence.

**Methods:**

A retrospective analysis of the records of all patients undergoing treatment in this institution between 2001 and 2007 (*n* = 2510) has been performed. We evaluated demographic data, reason for contacting the unit, the referring person or institution, the person or institution in charge of the care and custody of the child, the number of contacts with the clinic, presence of physical or psychiatric illness of a parent, and medications prescribed.

**Results:**

Ages of patients ranged from 1 to 17. Gender distribution was even. Having experienced the death of a relative, experienced violence, or having witnessed traumatic death were the main reasons for presentation. The utilization rates of immigrants rose throughout the observation period. Children from foster care were seen less frequently than expected. Medication was hardly prescribed.

**Conclusions:**

Ample utilization of this institution clearly demonstrates the need for short-term acute outpatient trauma therapy for children and adolescents. Efforts to provide easily accessible institutions for youth who experience traumatic events should be stepped up.

## Abstract (de)

**Grundlagen:**

Diese Untersuchung hatte zum Ziel, die Inanspruchnahme einer Ambulanz, welche niedrigschwellige, Trauma-fokussierte Kurzpsychotherapie für Kinder und Jugendliche anbietet, während der ersten sechs Jahre ihres Bestehens zu untersuchen.

**Methodik:**

Wir führten eine retrospektive Analyse der Krankengeschichten aller zwischen 2001 und 2007 behandelten Patienten (*n* = 2510) durch und dokumentierten demographische Daten, Gründe für die Behandlung, zuweisende Institution, Obsorge berechtigte Person oder Institution, die Anzahl der Kontakte, psychische oder körperliche Erkrankung eines Elternteils und verschriebene Medikation.

**Ergebnisse:**

Die behandelten Patienten waren zwischen 1 und 17 Jahre alt, die Geschlechterverteilung war gleichmäßig. Häufigster Anlass für eine Kontaktaufnahme waren der Tod eines Verwandten, die Tatsache, Zeuge eines gewaltsamen Todes geworden zu sein oder andere Gewalterfahrungen. Die Inanspruchnahme durch Migranten stieg während des Untersuchungszeitraumes laufend an. Kinder aus Pflegefamilien suchten die Institution seltener als erwartet auf. Medikation wurde kaum verschrieben.

**Schlussfolgerungen:**

Die intensive Nutzung dieser Institution zeigt deutlich den großen Bedarf an kurzfristiger akuter Traumatherapie für Kinder und Jugendliche. Bemühungen, leicht zugängliche Institutionen für traumatisierte Kinder und Jugendliche zur Verfügung zu stellen, sollten intensiviert werden.

## Introduction

Psychiatric trauma is defined as an experience that is emotionally painful, distressful, or shocking and which may result in lasting mental and physical deficits. Different kinds of psychiatric trauma include physical, emotional or sexual abuse, experience of loss, neglect, war, terrorism, natural disasters, and violence within families, communities, or schools.

Unfortunately, the experience of trauma during childhood is a common occurrence. Millions of children in Europe and the USA encounter traumatic events every year [1–3]. The number of unreported cases is unknown, but it has been estimated that one in ten children experience maltreatment [4]. Austria is a country with 8.4 million inhabitants of which almost 20 % are 19 years and younger [5]. Given the fact, that 9–22 % of all girls and 5–15 % of all boys will, at some time during childhood and adolescence, experience sexual assault or sexual harassment it has to be assumed that around 160,00 girls and 80,000 will be victims of sexual abuse. Approximately one fifth of Austrian adolescents seem to be affected by mental health problems [6].

Among the possible consequences of trauma exposure are negative health outcomes, such as post-traumatic stress disorder (PTSD), depression, substance abuse, and chronic illnesses [2, 3]. Experiencing domestic violence or the death of a parent during childhood not only predisposes a child to developing PTSD and depressive disorders later in life [7, 8] but also has been linked to a high risk of drug addiction and various somatic diseases, thereby increasing the risk for many of the leading causes of mortality in adults [9–11]. Moreover losing a parent early in life has been identified as an independent risk factor for additional trauma exposure [12], and a growing body of evidence suggests an association between early adverse events and an increased prevalence of subclinical psychotic phenomena [13]. Although there is a high degree of variability in responses to traumatic events that remains unexplained by psychological, biological, or genetic factors, there is strong evidence that early intervention as well as the perception of social support is a predictor of a lower rate of trauma-related symptoms [14–16]. Early contact between specialists and traumatized children is important in order for clinicians to identify the children’s needs and support their psychological and physical recovery [17].

Even though the detrimental effects of early childhood trauma on development are well established and if untreated can even persist well past the traumatic experience, there is little done with regards to public policy to address the urgent needs of this population and outpatient facilities for mentally disordered minors are scarce [18]. As such the authors have chosen to examine the use of an existing trauma intervention resource to see whether such a resource indeed fulfills these needs.

Given the large number of traumatized youth and the need for early intervention within this population, the current study examined the utilization of an outpatient clinic in Vienna, Austria, called “Die Boje” (“The Buoy”; http://www.die-boje.at/), which provides crisis intervention and short-term psychotherapy for children and adolescents who have experienced traumatic events. We investigated the type of care provided by the clinic and its patient population during its first 4 years of operation, in order to better understand the community’s needs for acute psychotherapeutic intervention for traumatized youth.

In Austria, health insurance is mandatory for everyone and The Buoy is accessible to every person with health insurance, so we expected an unbiased cross section through the population in need of a suchlike service.

Currently, The Buoy treats approximately 1000 outpatients per year [19]. Of these, about 25 % are affected by parental loss through suicide, accident, disaster, or illness. Another 30 % have experienced physical and/or psychological violence. The annual treatment setting encompasses around 7000 treatment hours with psychological, psychotherapeutic, and psychiatric modules. Children usually attend the clinic shortly after a traumatic event has occurred and are offered a package of up to 10 h of crisis intervention in a phase-oriented approach based on recent literature [20]. The average number of contacts is about seven, which confirms the short-term support nature of the institution. If necessary, patients are transferred to an institution capable of offering long-term treatment. In some cases, children are supported on a more long-term basis at the outpatient clinic itself.

## Methods

We retrospectively reviewed the records of each patient that contacted The Buoy in a 6-year-period between its opening in December 2001 and December 2007, including demographic, familial, and trauma-related information. We documented sex, date of birth, country of origin, date and age at first contact, reason for contacting the unit, the referring person or institution, the person or institution in charge of the care and custody of the child, the number of contacts with the clinic, presence of physical or psychiatric illness of a parent, and medications prescribed.

The study was approved by the Ethics Committee of the Medical University of Vienna (EK 841/2011).

## Results

During the 6-year-examination-period, a total of 2510 people sought help at The Buoy, with a balanced gender distribution of 1304 (51.9 %) boys and 1206 (48.1 %) girls. The number of patients frequenting the clinic consistently increased over time. While there was only one patient in 2001, 83 children were treated in 2002. In 2003 and 2004 numbers increased to 335 and 365 patients, respectively and rose again in 2005, when 632 patients contacted the clinic for help. A decline was observed during the following years with 550 treated in 2006 and 540 patients in 2007.

The age at first contact ranged from 1 to 17 years, with a peak between ages 8 to 11 and a mean age of 10.3 years and (SD 4.1) median of 10 years. Only 501 patients were younger than 7 years.

The predominant reasons for consultation included death of a parent (*n* = 517; 20.6 %), witnessing violent death (*n* = 272; 10.5 %) being either murder and/or suicide (*n* = 188; 7.5 %) or death by accident (*n* = 84; 3.4 %), having a severely somatically (*n* = 209; 8.3 %) or mentally ill (*n* = 173; 6.0 %) relative, experiencing continuous physical or psychological maltreatment (*n* = 299; 11.9 %), experiencing a traumatic divorce of the parents (*n* = 242; 9.7 %), or exhibiting conduct disorders in connection to traumatic experience (*n* = 403, 16,0 %). In 506 cases (31.8 %) one or both parents were mentally ill, in 273 (17.9 %) cases severe somatic illness was present in one or both parents, and 73 (2.9 %) had at least one parent who was both mentally and somatically ill. Three of the four 1-year-olds had witnessed the death of a relative, and one had a severely ill parent. Most of the 2-year-olds had also witnessed one of their parents dying or had at least one severely mentally or somatically ill parent.

Most patients (*n* = 1926; 81.4 %) were Austrian citizens, 86 patients originated from Turkey, 81 from Chechenia, 74 from what was the former Federal Republic of Yugoslavia, 20 from Poland, 13 from Iran, and the remainder were from other countries such as Afghanistan, Egypt, Slovakia, or Romania. The rate of non-Austrian citizens however continuously rose during the observation period. While during the first years only around 10 % of all patients were immigrants, children of immigrants represented 20 % of the clientele in 2007. Most patients (*n* = 2268; 90.4 %) lived in Vienna, 270 patients resided in neighboring counties, and 58 patients lived more than 100 km away from Vienna.

The average number of consultations was 10.2 (SD), the number of contacts ranging from 1 to 186 and a median of 4. Most patients (*n* = 682; 28.8 %) had not been referred to the center but rather found out about it by advertisements or word of mouth. In cases of referral, patients were sent by a “crisis intervention center”, a drop-in center for people experiencing acute crisis situations (*n* = 225; 10.8 %), psychologists or social workers (*n* = 309; 13.0 %), the Youth Welfare Service (*n* = 337; 14.2 %), school or nursery (*n* = 287; 12.1 %), the Department for Child and Youth Psychiatry of the Medical University Hospital (*n* = 231; 9.8 %), a physician (*n* = 198; 8.4 %), or an outpatient facility for developmental aspects.

Most patients did not receive any medication. In those cases where drugs were prescribed (*n* = 66; 2.6 %), they were mainly antidepressants, stimulants, or neuroleptics. Benzodiazepines were prescribed in one case only. Of all children, 12.3 % contacted the institution anew after the end of therapy. Custody was with the parents in most cases (both parents: 38.1 %, mother: 44.1 %, father: 10.4 %) with other family members in 6.0 % of all cases and with the Youth Welfare Service or foster families in 1.4 %. In almost half of the cases, the first appointment was held shortly after the occurrence of a traumatic event (*n* = 1149; 45.8 %). In 731 (29.1 %) of all cases, a single traumatic event had taken place some time ago and in 681 (27.1 %) cases children had been affected by continuous traumatization.

If reported measures do not sum up to 2510, missing data account for the difference.

## Discussion

Evaluation of the utilization of the institution “The Buoy” clearly showed that the demand for acute psychotherapeutic intervention for traumatized children and adolescents is obviously huge in the investigated area. Within a little over 6 years, the number of youth newly contacting the institution increased from 83 to more than 600 per year in 2005. The drop in patients during the years 2006 and 2007 has to be explained by the fact that funding of the institution has not been assured during that period, so treatments offered had to be restricted and demands could not be met. During the following years numbers rose again and currently remain steady around 1000 children that are treated per year [19]. Age distribution was rather even, still we were surprised by the fact that even one- and two-year-olds were seen in the clinic, indicating that the need for acute intervention for psychiatric trauma is not limited to older children or adolescents. While the average number of sessions was seven, the median was as low as four. We assume that, apart from the fact, that therapy offered by “Die Boje” is intended to be short-time psychotherapeutic intervention, some children will have been sent on to other institutions after one or two appointments.

The most frequent cause for trauma was witnessing or encountering the death of a parent or relative. This might be due to the fact that some of the therapists working at this clinic specialize in grief therapy; this information may have circulated within the community. Unfortunately core data did not allow differentiation between murder and suicide; given the fact that during the observation period suicide was around 35 times more frequent than murder both in Austria and in Vienna [5], we can assume that children had far more often witnessed a relative’s suicide than the murder of a close one.

The scarce administration of medication in the clinic is most likely due to the fact that during the observation period only one psychiatrist was working in the institution while all other therapists were psychologists. The limited medication administration may also indicate that the children and adolescents being treated in the clinic had, on the whole, been mentally healthy or at least not been in treatment for serious conditions before the occurrence of the traumatic event.

In the first years of “The Buoy’s” existence, almost 90 % of all patients were Austrian citizens. During 2006 and 2007 the percentage of immigrants rose to more than 20 %. In 2014, 30 % of all subjects treated at the institution were non-Austrian citizens [19]. Given the fact that 21.7 % of all inhabitants of Vienna are non-Austrian citizens [5] it is obvious that, at first, efforts by “The Buoy” to reach immigrant groups were not sufficiently effective. At the same time it is known that personal migration in childhood may pose significant developmental challenges and increases the predisposition for developing mental disorders; all the while these risks are complicated by the fact that migrant families often have limited access to mental health-care institutions [20–23]. We are unable to determine what specific barriers may have prevented service utilization in this case but have to assume that access to information about mental health-care institutions is limited in this group. Given that motivation and maintenance of therapy is traditionally underrepresented in families of immigrant children, initiation of compliance is a major challenge for many community-based mental health clinics [24]. Special attention hence ought to be paid to this issue in the future development, organization, and administration of the focus on migrants in such institutions [25].

Youth in foster care or under the custody of the Youth Welfare Service, who generally represent a highly traumatized population [26], were also underrepresented in our sample, comprising only 1.4 % of all patients. The most likely explanation for their relative absence from our patient group is the fact that many institutions offering foster care already have established relations with various psychiatric services. As such, psychotherapy might be organized by those institutions.

Research on resilience, an individual’s ability to properly adapt to stress and adversity, clearly shows that in addition to individual factors and familial circumstances, favorable social environments and perceived support in stressful situations play important roles in fostering mental health [21, 27–29]. This highlights the value of a community-based outpatient clinic that provides acute and crisis interventional treatment for children and adolescents, particularly given, that the stigma of mental illness may prevent many parents from taking their child to a hospital’s department of child and adolescent psychiatry. Availability of low-threshold psychotherapeutic “first-aid” outpatient facilities for traumatized youth is of the utmost importance. These services may be especially vital for youth from disadvantaged backgrounds, since low socioeconomic status is associated with limited access to family advice and lack of access to common beneficial community resources [30, 31].

It is well known that individuals with histories of childhood trauma are significantly more likely than their non-traumatized counterparts to make use of mental health services, child care, and social services when older [32, 33]. Although research on developmental aspects concerning “windows of vulnerability” during childhood and adolescence is still scarce, it appears that the younger the children are when they experience traumatic events, the higher their risk is for developing adulthood mental health problems [34]. Intervening as early as possible clearly reduces the occurrence of mental health problems and mental disorders later in life [14, 15], which further supports the value of a clinic such as The Buoy. By providing information about psychiatric conditions and psychoeducation, outpatient clinics that target youth can also help reduce stigma and treatment barriers among adolescents [35].

## Limitations

The major limitations of the current study are its retrospective nature and the consequent lack of information on further development of these children and adolescents.

## Conclusion

This evaluation demonstrates a real need for trauma “first aid therapy” that had obviously not being met by other child and adolescent psychiatric services in the respective area. There is a great need for trauma therapy among youth and early intervening trauma therapy can, as is known, minimize the possibility of mental health problems later in life. Consequently, public policy efforts to provide easily accessible interventions for children who experience traumatic events should be bolstered.
